# Viral cystatin evolution and three-dimensional structure modelling: A case of directional selection acting on a viral protein involved in a host-parasitoid interaction

**DOI:** 10.1186/1741-7007-6-38

**Published:** 2008-09-10

**Authors:** Céline Serbielle, Shafinaz Chowdhury, Samuel Pichon, Stéphane Dupas, Jérôme Lesobre, Enrico O Purisima, Jean-Michel Drezen, Elisabeth Huguet

**Affiliations:** 1 Institut de Recherche sur la Biologie de l'Insecte, UMR CNRS 6035, Faculté des Sciences et Techniques, Parc de Grandmont, 37200 Tours, France; 2 National Research Council Canada, Biotechnology Research Institute, Montreal, Canada; 3 Institut de Recherche pour le Développement, Quito, Ecuador; 4 Laboratoire Ecologie Evolution Symbiose, UMR 6556, Université de Poitiers, Poitiers, France

## Abstract

**Background:**

In pathogens, certain genes encoding proteins that directly interact with host defences coevolve with their host and are subject to positive selection. In the lepidopteran host-wasp parasitoid system, one of the most original strategies developed by the wasps to defeat host defences is the injection of a symbiotic polydnavirus at the same time as the wasp eggs. The virus is essential for wasp parasitism success since viral gene expression alters the immune system and development of the host. As a wasp mutualist symbiont, the virus is expected to exhibit a reduction in genome complexity and evolve under wasp phyletic constraints. However, as a lepidopteran host pathogenic symbiont, the virus is likely undergoing strong selective pressures for the acquisition of new functions by gene acquisition or duplication. To understand the constraints imposed by this particular system on virus evolution, we studied a polydnavirus gene family encoding cyteine protease inhibitors of the cystatin superfamily.

**Results:**

We show that *cystatins *are the first bracovirus genes proven to be subject to strong positive selection within a host-parasitoid system. A generated three-dimensional model of *Cotesia congregata *bracovirus cystatin 1 provides a powerful framework to position positively selected residues and reveal that they are concentrated in the vicinity of actives sites which interact with cysteine proteases directly. In addition, phylogenetic analyses reveal two different *cystatin *forms which evolved under different selective constraints and are characterized by independent adaptive duplication events.

**Conclusion:**

Positive selection acts to maintain *cystatin *gene duplications and induces directional divergence presumably to ensure the presence of efficient and adapted cystatin forms. Directional selection has acted on key cystatin active sites, suggesting that cystatins coevolve with their host target. We can strongly suggest that cystatins constitute major virulence factors, as was already proposed in previous functional studies.

## Background

In a host-parasite interaction the associated partners can have an influence on each other's evolution [1]. Molecular signatures of these complex evolutionary processes can be detected in the genomes of both organisms involved in such associations. Indeed, genes encoding pathogenicity factors directly involved in counteracting host defences or vice versa are expected to be subject to positive selection, driven by an arms race between the two partners. Such coevolutionary processes have been well described in certain plant-pathogen interactions, where the host resistance genes and corresponding avirulence genes in the pathogen show evidence of positive selection [2]. In the *Xanthomonas*-pepper interaction, the Hrp pilus, a filamentous structure allowing bacteria to directly inject toxins into plant cells, also evolves under positive selection, thereby avoiding the plant defence surveillance system [3]. Positive selection has also been detected in insect-pathogen interactions. For example, in *Drosophila*, RNA interference (RNAi) molecules involved in anti-viral defence are among the fastest evolving genes in this insect. This rapid evolution is due to strong positive selection, illustrating that the host pathogen arms race between RNA viruses and host antiviral RNAi genes is very active and significant in shaping RNAi function [4].

We are interested in characterizing the evolutionary processes underlying the insect host-parasite interactions between lepidopteran hosts and parasitoid wasps. In these systems, the endoparasitoid wasp larvae develop inside the lepidopteran host despite the hostile environment this habitat represents. One of the most original strategies developed by these wasps to defeat these defences is the injection of a symbiotic polydnavirus (PDV) at the same time as the wasp eggs [5-7]. PDVs are divided in two genera, ichnoviruses and bracoviruses, which are associated with tens of thousands of endoparasitoid wasps belonging to two different families, Ichneumonidae and Braconidae [8]. PDVs are found in these wasps as proviruses which are transmitted vertically from one wasp generation to the next [9-13]. Proviruses are excised from the wasp genome in the female ovaries and, after replication, are injected into the host caterpillar as multiple double-stranded DNA circles packaged in capsids. The virus does not replicate in the host caterpillar, but viral gene expression and protein production are essential for alterations to the immune system and development of the host leading to successful development of the wasp larvae.

In this biological system, the virus plays key roles both in the mutualistic association with the wasp and in the parasitic association between the wasp and the caterpillar. PDVs are therefore likely to display molecular signatures which reflect constraints imposed both by the wasp and the host caterpillar. So far, however, reports have principally concentrated on the influence of wasp evolution on viral genomes. Braconid wasps carrying PDV form a monophyletic lineage, suggesting a unique event of association between the wasp ancestor and the virus ancestor and a vertical transmission of the virus along wasp lineages [14]. Accordingly, a phylogenetic study of *Cotesia *spp. and their associated viruses has shown a codivergence between the two mutualists [15]. Finally, recent data on the genome sequence of several PDVs has revealed that these viruses harbour a large number of eukaryotic genes likely picked up from the wasp genomes. These genes form multigene families that are good candidates to be involved in alteration of host caterpillar physiology [16-20]. Surprisingly, very few studies have focused on the potential influence of the host caterpillar on viral gene evolution despite the strong selective pressure this habitat represents. In this paper, we report on the molecular evolution of a viral gene family considering both wasp evolution and the selective pressure imposed by the caterpillar hosts.

Our model system is the interaction between the braconid wasp *Cotesia congregata *and its lepidopteran host, the tobacco hornworm, *Manduca sexta*. The PDV associated with *C. congregata *(CcBracovirus, CcBV) has been sequenced, revealing the presence of numerous genes possibly involved in host deregulation [20]. Among these viral genes, one gene family encoding cystatins constitutes an interesting candidate system to study the influence of the host-parasitoid association at the viral molecular level. Cystatins are tightly binding reversible inhibitors of papain-like cysteine proteases, and are widespread in plants and animals [21]. They are characterized by three conserved domains forming the site of interaction with C1 cysteine proteases: an N-terminal glycine, a glutamine-X-valine-X-glycine motif and a C-terminal proline-tryptophane amino acid pair [22,23]. Cystatins and their target proteases have often been shown to be involved in host-parasite interactions with cystatins either playing the role of defence molecules or virulence factors. For example, in parasitic nematodes, cystatins are thought to play a key role in controlling the host immune response [24-26]. Remarkably, plant cystatins acting as defence proteins have been shown to evolve under strong positive selection in response to cysteine proteases released by phytophagous insects. In this system, it has been suggested that plant cystatins and insect cysteine proteases are involved in a coevolutionary process [27].

CcBV *cystatins *constitute the first description of *cystatin *genes in a virus and are organized in a multigene family, composed of three genes present on the same circle [17,20]. To date, there is no evidence of *cystatin *genes in *Microplitis demolitor *bracovirus (MdBV) which has been fully sequenced [19] and they have only been identified in one other PDV (GiBV) from the braconid wasp *Glyptapanteles indiensis *[13]. Both genomic and physiological features of cystatins suggest that these viral proteins could play an important role in the host-parasite association. First, the genomic organization in a multigene family could be indicative of selective pressures acting on these genes. Indeed, Francino [28] suggested that gene duplications that can lead to an increase in protein dosage are favoured by selective pressures. Second, *cystatin *genes are expressed rapidly and at an extremely high level during parasitism. This early and prolonged expression could be indicative of a role of cystatins in the early steps of host physiological disruption, as well as in the maintenance of this perturbed state. Finally a recombinant viral cystatin (Cystatin 1) was shown to be a functional and specific cysteine protease inhibitor [17].

In this study we checked for molecular signatures associated with positive selection that may act on the viral *cystatin *gene family. We demonstrate strong and lineage-specific adaptive evolution acting on these genes. Using homology modelling and molecular dynamics (MD) simulation techniques we obtained the three-dimensional (3D) structure of CcBV cystatin 1. The predicted model of the 3D structure of CcBV cystatin provides a framework to position the positively selected residues, and reveals that these are situated in key sites which are important for the interaction with target proteases. This particular selection, which is probably imposed by host defences, emphasizes the potential role of cystatins as pathogenic factors and suggests that cystatins coevolve with host cysteine proteases.

## Results

### *Cystatin *genes from PDVs associated with *Cotesia *species exhibit weak genetic divergence

To study the molecular evolution of viral *cystatin *genes, we isolated 48 sequences from PDVs associated with nine *Cotesia *species, revealing that several *cystatin *forms exist in the same species. Accession numbers are provided in Additional file 1. The divergence of the third domain prevented amplification of this region, therefore the *cystatin *sequences isolated contain only the first two interactive sites. It is extremely unlikely that endogenous wasp cystatins could be amplified by this approach given that PDV cystatins show a low level of relatedness to insect cystatins, and are no more related to insect cystatins than to mammalian inhibitors [17].

Four alleles isolated from *C. glomerata *correspond to a pseudogene with a stop codon situated in the same position for all sequences obtained. Genetic divergence estimated by pairwise distance, which gives the mean number of substitutions per site, ranges from 0.007 to 0.31; these weak values suggest that *cystatin *genes are very similar. Finally, genetic algorithm recombination detection (GARD) detected no evidence of recombination, allowing us to estimate phylogenies and test for positive selection on *cystatin *genes.

### *Cystatin *phylogeny shows two main *cystatin *forms

**Figure 1:**
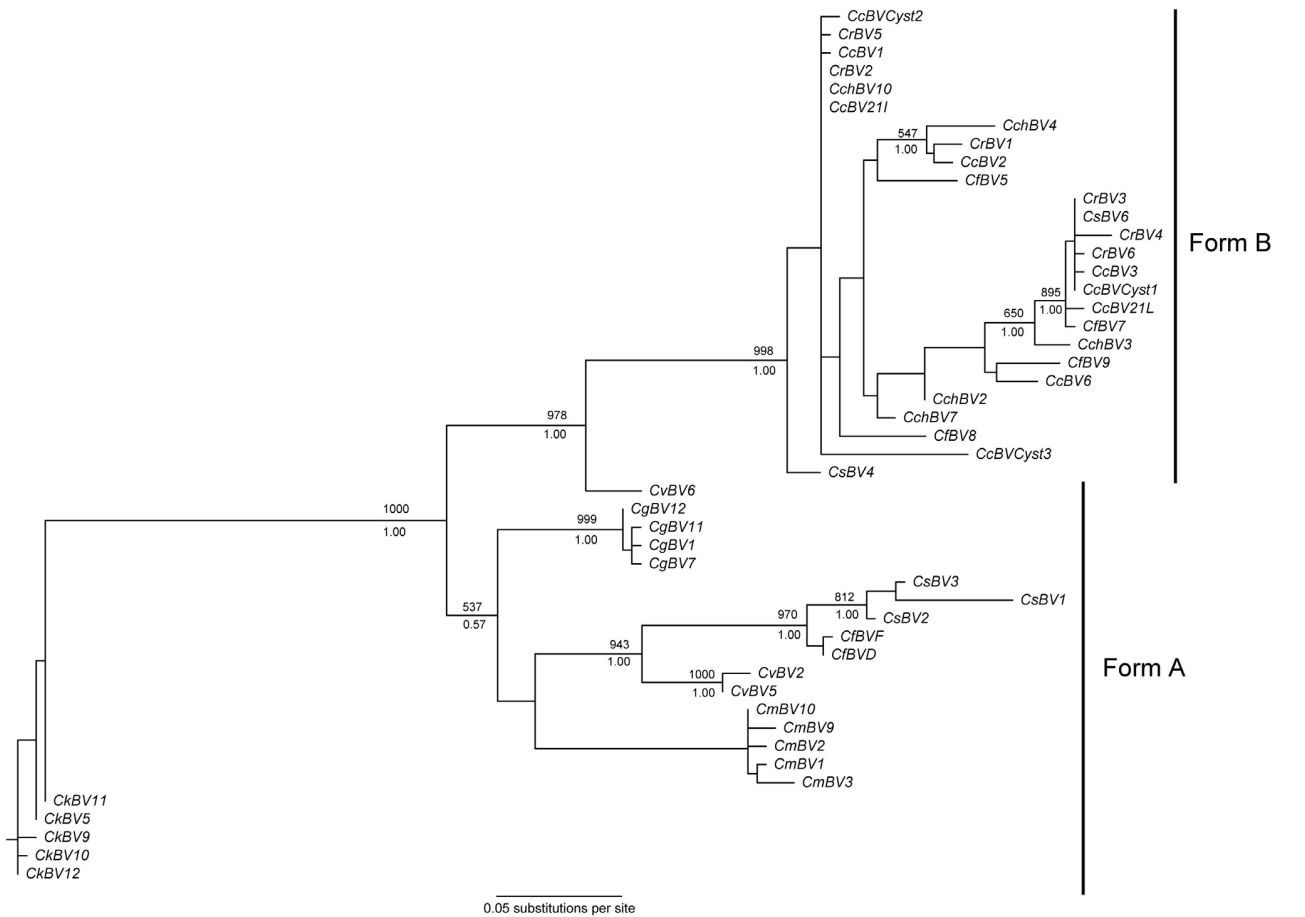
**Cystatin gene tree obtained by maximum likelihood**. Node supports are shown by bootstraps and by posterior probabilities from Bayesian inferences above and below each branch, respectively. Bootstrap scores or posterior probabilities lower than 50% are not represented. Sequences were obtained from bracoviruses of *Cotesia congregata *(CcBV), *Cotesia flavipes *(CfBV), *Cotesia chilonis *(CchBV), *Cotesia melanoscela *(CmBV), *Cotesia vestalis *(CvBV), *Cotesia rubecula *(CrBV), *Cotesia sesamiae *(CsBV), *Cotesia karyai *(CkBV)*and Cotesia glomerata *(CgBV). Cystatin sequences from *CcBV *genome are noted *CcBV*cyst1, *CcBV*cyst2 and *CcBV*cyst3.

*Cystatin *phylogeny was studied using Bayesian inference and maximum likelihood analysis. Both methods gave the same tree topology. The best tree obtained by maximum likelihood is presented in Figure 1 with bootstrap scores and posterior probabilities. The tree presented was unrooted because there is no suitable outgroup for this study. Phylogenetic analysis revealed the presence of two major cystatin forms supported by high bootstraps and posterior probabilities. The form A cystatins are constituted by *CkBV*, *CmBV*, *CgBV*, *CvBV *sequences and *CfBV*D and F, *CsBV*1, 2 and 3. The form B cystatins are constituted by *CcBV*, *CchBV*, *CrBV *sequences and *CfBV *5, 7, 8 and 9, *CsBV*4 and 6. The form A, in which each clade is supported by high scores, matches the wasp phylogeny [29]. Indeed, in this case, *cystatin *sequences from a same species group together in the same way as in the wasp phylogeny [29]. There is one exception for the *CvBV*6 sequence which is not grouped with other *C. vestalis *virus sequences. In form B the organization is different and does not match wasp phylogeny [29]. Indeed, we do not find a preferential association between sequences from the same wasp species, and the internal branches of this clade are not well supported. The second important difference concerns the branch length: form A *cystatins *exhibit higher overall branch lengths than form B, suggesting different rates of evolution for these two cystatin forms. This phylogeny suggests the existence of two main ancestral *cystatin *gene forms which have evolved under different constraints to give form A and B. Indeed in form A *cystatins*, long branch lengths are exhibited and follow wasp speciation, as opposed to the form B *cystatins*, which exhibit shorter branch lengths and seem to evolve independently of the wasp speciation process.

Among *cystatin *sequences isolated from a same species some are likely to correspond to allelic forms such as *CgBV cystatin *sequences whereas others seem to be different *cystatin *copies such as *CsBV*1, *CsBV*2 *and CsBV*3 (form A). *Cystatin *copies obtained from the CcBV genome sequencing project (*CcBV*cyst1, *CcBV*cyst2 and *CcBV*cyst3) are found in form B and therefore do not seem to have any orthologous sequences in *Cotesia melanoscela*, *Cotesia glomerata *and *Cotesia kariyai *bracoviruses. In form B *cystatins*, these three *cystatin *copies are not grouped together, indicating that duplications occurred before or at the same time as wasp speciation. In contrast, *cystatin *copies or *cystatin *alleles in form A are grouped by wasp species, suggesting that duplications occurred after wasp speciation.

### *Cystatin *genes evolve under positive selection

In order to analyse protein evolution and test for positive selection in *cystatins*, the maximum likelihoods of different substitution models were determined and compared using chi-squared statistics. Model M0 assumes that all sites have the same ω value whereas M3 distributes amino acids into three classes allowing sites to evolve under different evolutionary constraints. M8A model constrains amino acids to have ω values of at most 1 whereas the M8 model adds a supplementary class of ω allowing sites to evolve under positive selection. Likelihood ratio tests (LRTs) indicated that selected models M3 and M8 fit the data better than M0 and M8A, respectively, with *P*-values of less than 0.001 (Table 1). These results suggest firstly that all amino acids are not constrained by the same selective pressures and secondly that cystatin sequences, with an average ω value of 1.2 over all sites and branches, evolve under positive selection. A class-specific site selection analysis was performed to determine the heterogeneity of selection regimes relative to the amino acid position. This analysis indicated that more than 30% of all amino acids are under strong diversifying selection (Table 1).

**Table 1:** Positive selection analysis among sites and lineages of viral cystatins from *Cotesia *spp. parasitoid wasps

**Site analysis**						
Models	χ^2 ^value	df	*P*-value for best model	Global ω	ω > 1, parameters	PSS

M0 versus M3	125.00	6	< 0.001	1.31	ω = 2.65, *p *= 0.310	26, 5*, 11**
M8A versus M8	19.57	1	< 0.001	1.21	ω = 2.85, *p *= 0.289	26, 7*, 5**

**Branch site analysis**						

Models	χ^2 ^value	df	*P*-value for best model		ω > 1, parameters (foreground lineage)	

M1a versus MA	126.81	2	< 0.01		ω = 11.93, *p *= 0.232	
M3 versus MB	73.20	3	< 0.01		ω = 12.04, *p *= 0.233	

Notes: df is degrees of freedom; PSS is the number of positive selected sites; * corresponds to a posterior probability greater than 95% of having ω > 1 and ** corresponds to a posterior probability greater than 99% of having ω > 1.

### Modelling by MD simulations reveals that the overall folding of known cystatin structures are preserved in CcBV cystatin 1

We wanted to determine whether PDV cystatins adopt a similar 3D structure to chicken cystatin and human cystatins for which the 3D structures have been resolved by crystallography [22,23,30,31]. This constitutes an important prerequisite to be able to interpret the potential consequences of the position of the positively selected sites with respect to the function and the evolution of function of PDV cystatins.

**Figure 2:**
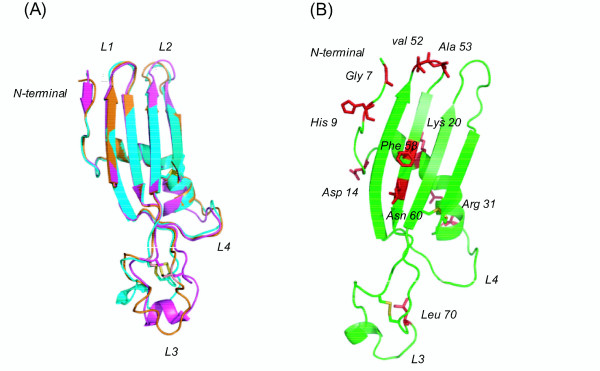
**Molecular model of CcBV cystatin 1 obtained from the average structure**. (A) The superimposed molecular dynamics (MD) average structure of CcBV cystatin 1 orange (1–5 ns), cyan (5–7 ns) and purple (8–10 ns) of 10 ns MD simulation trajectory. (B) Positively selected residues (probability 95%) are represented as a red colour capped stick model on the secondary structure (green) of the final model of CcBV cystatin 1 average structure (1–10 ns). Glycine in N-terminal and Valine and Alanine in the L1 are important for C1 protease binding. CcBV mature cystatin 1 amino acid numbering is used.

In a previous study, a multiple sequence alignment of CcBV cystatin 1 was performed with insect, chicken, mouse and human cystatins [17]. Although there is only a modest level of sequence identity among CcBV cystatin 1, human cystatin D and chicken egg white cystatin, a reasonable alignment could be found that permitted a homology model to be built. A 10 ns MD simulation was carried out to check the stability of the modelled structure. The energy of the system levelled off after about 800 ps, indicating that an equilibrium state had been reached (data not shown). The overall structure was stable during the simulation. Visual inspection of the trajectory showed that the global fold remained essentially intact. The PROCHECK program [32] did not flag any conformational problems with the structure. Figure 2A shows a superposition of three average structures during three different time frames in the trajectory. We see that the structures of L1, L2 and L4 are very stable during the simulation. L3 shows somewhat greater structural variability.

The modelled structure preserves the overall fold of solved cystatin structures: a five-stranded anti-parallel β-sheet wrapped around a five-turn α-helix (Figure 2). However, β1 maintains its beta strand conformation for only part of the MD simulation. The protease binding site shows a wedge-shaped area formed by N-terminal residues (Glycine 6), the first hairpin loop L1 (QxVxG motif positions 50 to 54) and the second hairpin loop L2 (PW). The two conserved type 2 cystatin disulfide bonds are also preserved in this 3D model of CcBV cystatin 1. Importantly, the 3D model shows that the three conserved domains in CcBV cystatin 1 form the typical tripartite 'wedge' which was shown in the crystal structure of human cystatin B in complex with papain to slot into the protease's active site [23]. These domains therefore display a correct conformation in CcBV cystatin 1, consistent with previous data showing that cystatin 1 is a functional cysteine protease inhibitor [17].

### Most positively selected sites are situated in the vicinity of the cystatin active sites

**Figure 3:**
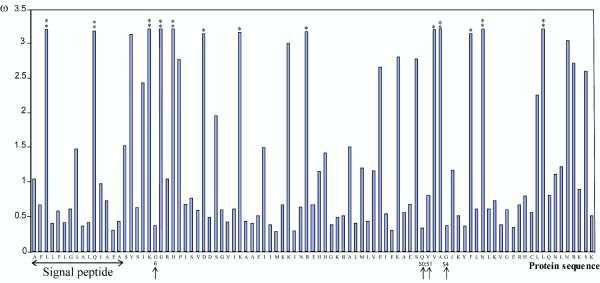
**Graphic representation of variable selective pressures (ω) along the protein sequence**. The * indicates a posterior probability greater than 95% of having ω > 1 and ** indicates a posterior probability greater than 99% of having ω > 1. Conserved amino acids implicated in the interaction with target proteases are indicated by arrows and are numbered according to the mature protein.

Sites showing a significant probability (greater than 95%) of being positively selected in viral cystatins were mapped onto the primary sequence (Figure 3) and on the structural model of CcBV cystatin 1 (Figure 2B). Out of the 12 positively selected sites identified in the mature protein, four are situated in the N-terminal segment containing the conserved Glycine 6 residue (residues Lysine 5, Glycine 7, Histidine 9 and Aspartic acid 14) and two residues are within the first hairpin loop L1 containing the QxVxG motif (residues Valine 52, Alanine 53) (Figures 2B and 3). Lysine 20 and Arginine 31 are located in the α-helix and Phenylalanine 58 and Asparagine 60 at the β3 sheet. Leucine 70 is located at loop 3 between β3 and β4 near the first disulfide bond.

Analysis of the viral cystatin protein alignment among the different wasp species revealed that out of the 12 positively selected sites, 8 (corresponding to Lysine 5, Glycine 7, Histidine 9, Aspartic Acid 14, Lysine 20, Arginine 31, Asparagine 60 and Leucine 70) undergo radical changes in biochemical properties which could induce changes in protein conformation and specificity (see Additional file 2 and Additional file 3). For example, Lysine 5, which is a polar and hydrophilic amino acid, can be replaced in other viral cystatin lineages by a leucine which is a hydrophobic residue.

Two amino acids under strong positive selection are also found in the signal peptide. These residues are located in the central, commonly hydrophobic part of the signal peptide, and they do not undergo changes in hydrophobicity. Although selection on signal peptides has rarely been analysed it has already been described in virulence proteins [33] and it is thought that variations in the signal peptide could affect exportation of proteins [34,35]. In our biological system, viral cystatins are secreted by the host secretory system, therefore we could speculate that the modification of the signal peptide composition could ensure more efficient secretion.

### Two main *cystatin *lineages show different evolutionary histories

To test for evidence of positive selection among lineages we performed a branch site analysis. In MA and MB models we assigned a value of ω ≤ 1 (ω_0_) for form A *cystatins *(background branches) which is congruent with the wasp tree and should evolve under purifying selection and a value of ω > 1 (ω_1_) for form B *cystatins *(foreground branches) which is not congruent with wasp phylogeny and therefore should evolve under positive selection. LRTs indicate that MA and MB fit the data better than models M1a and M3, respectively, with *P*-values of less than 0.01. Furthermore, these analyses suggest that in foreground lineages about 23% of sites evolve under strong positive selection with ω values of around 12 (Table 1). Branch-site analysis results therefore suggest that form A *cystatins *are mainly undergoing purifying selection, whereas form B *cystatins *are mainly evolving under positive selection.

**Figure 4:**
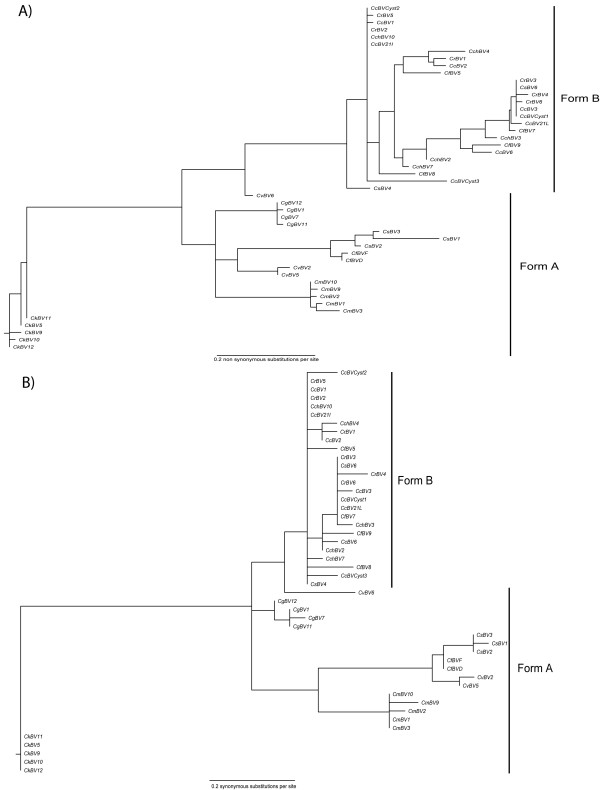
**Cystatin sequence tree under local substitution model**. (A) Tree scaled on expected number of nonsynonymous substitutions per site. (B) Tree scaled on expected number of synonymous substitutions per site.

As this phylogenetic analysis by maximum likelihood (PAML) analysis did not allow us to determine the nature of selective pressures acting on each branch, we constructed trees in which branch length represents the expected number of substitutions per codon. The tree in Figure 4A is based on nonsynonymous substitutions, whereas the tree in Figure 4B represents the expected number of synonymous substitutions in *cystatins*. These representations showed a difference in the type of substitutions occurring in the two *cystatin *forms and suggested that divergence in form B is principally explained by nonsynonymous substitutions between *cystatin *sequences and that divergence in form A *CmBV*, *CvBV*, *CfBV*, *CkBV *and *CsBV *sequences are particularly due to synonymous substitutions which occur principally in the internal branches. A similar analysis conducted with a nuclear wasp gene (COI) did not reveal differences in synonymous and nonsynonymous substitutions between wasp species (data not shown) suggesting that the different evolutionary patterns observed above are specific to viral cystatins.

**Figure 5:**
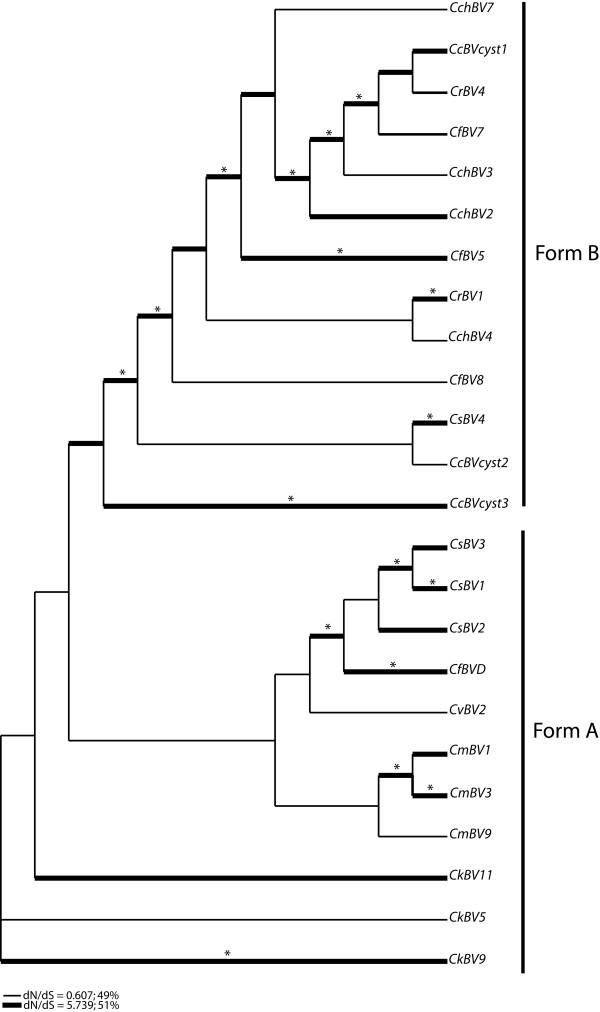
**Branches under positive selection estimated according to the genetic algorithm-branch analysis**. Percentages for branch classes in the legend reflect the proportion of total tree length (measured in expected substitutions per site per time unit) and evolving under the corresponding value of *d*_*N*_/*d*_*S*_. The * indicates a posterior probability greater than 95% of having ω > 1.

To gain further insight into the nature of selective pressures acting on each branch we performed a genetic algorithm (Ga)-branch analysis that confirmed that all lineages are not constrained by the same evolutionary forces. Ga-branch analysis selected a model with two classes of ω. In total, 49% of branches are assigned to a ω of 0.6 and 51% to a ω class of 5.7 (Figure 5). Both types of branches are present in form A and B, however their position in the tree differs. In form A, positive selection occurs in terminal branches between intraspecies *cystatin *copies. This analysis emphasizes that divergence between *cystatin *copies from the same wasp species occurred by positive selection. Internal branches in form A *cystatins *are characterized by purifying selection, indicating that *cystatin *genes evolved under conservative selection during wasp speciation. A different pattern is observed in form B *cystatins*, where positive selection occurred preferentially in internal branches of the tree. Indeed positive selection occurred in the original branch and in almost all internal branches of this clade, thereby diluting the effect of wasp speciation on *cystatin *divergence. In conclusion, PDV *cystatin *divergence has been driven by positive selection, which has acted at different levels, before, during or after the wasp speciation process.

## Discussion

### *Cystatin *genes constitute a young multigene family compared with the other *Cotesia *bracovirus genes

*Cystatin *genes appear to be unique compared with the other gene families found in the viruses associated with *Cotesia *genus. First *cystatin *divergence, which gives the mean number of substitutions per site, is very weak ranging from 0.007 to 0.31, whereas divergence between CcBV copies of other viral genes like protein tyrosine phosphatases (PTP) or IκB-like proteins range from 0.56 to 0.832 (see [36]). In contrast to PTP or IκB-like proteins, which are both widely distributed in the Bracoviruses carrying PDV [19], *cystatin *genes are so far restricted to *Glyptapanteles *and *Cotesia *[13,20]. Furthermore *G. indiensis cystatin *is found in a single copy, whereas three copies are found in *C. congregata *[13,20], suggesting that the *C. congregata cystatin *gene family resulted from a recent duplication event. The weak divergence between *cystatin *lineages as well as their narrow phylogenetic distribution constitute evidence of the recent acquisition of *cystatin *genes by the bracovirus.

As a consequence, studying *cystatin *gene evolution might allow us to understand the preliminary evolutionary processes involved in the diversification of a young multigene family. The recent events of acquisition and duplication of *cystatin *genes might explain the lack of divergence between *cystatin *copies and our inability to distinguish between orthologous and paralogous relationships between copies. For this reason, in our analysis all *cystatin *copies that might include orthologues and paralogues were analysed together.

### Are *cystatin *genes codivergent with wasp species?

PDVs are integrated into wasp chromosomal DNA as a provirus which is inherited exclusively in a Mendelian fashion [37]. There is no evidence that PDVs can be transferred horizontally between parasitoids and PDVs do not replicate in the host caterpillar. In view of this particular virus life-cycle we can hypothesize that PDV gene evolution is in part determined by evolutionary constraints acting on wasps, such as a phyletic constraints. Nevertheless, viral genes, which are likely to be involved in parasitism success, also have to adapt to caterpillar defences.

A study comparing wasp phylogeny of seven *Cotesia *species based on mitochondrial DNA and viral evolution using the CrV1 gene, has shown a perfect congruence between wasp and viral phylogenies [15]. In our study the *cystatin *gene tree also shows perfect codivergence between wasp and viral genes for some *cystatin *gene lineages. The evolution of these *cystatin *forms appears therefore to be constrained by wasp phylogeny and the molecular constraints acting on the wasp genome. However, in contrast to the results obtained using CrV1, not all *cystatin *lineages follow wasp evolution; instead, some *cystatin *genes are submitted to other constraints since their phylogeny does not match wasp phylogeny.

### *Cystatins *are under strong selective pressure acting on key sites

The study of selective pressures acting on *cystatin *genes confirms that *cystatin *genes are not simply constrained by wasp evolutionary history. Indeed, we showed that *cystatin *gene evolution is driven by a strong positive selection. The global ω value of 1.2 obtained through analysis of the viral *cystatins *is similar to the value obtained with plant cystatins [27]. Plant cystatins are involved in a plant-phytophagous interaction, but in that case cystatins play a role in defence against digestive cysteine proteases of herbivorous insects. Plant cystatins and their targets are thought to be involved in a coevolutionary process. Other examples of positive selection are also available with pathogen molecules. A previous study performed on an Ichnovirus protein involved in host immune inhibition has shown that positive selection was only detected at particular protein sites [38]. Our study constitutes the first example of a major impact of positive selection in the evolution of a bracovirus protein.

The identification of the position of positively selected sites in PDV cystatins in the primary sequence and in the 3D model revealed that 70% of sites are situated within or proximal to the N-terminal segment harbouring the conserved Glycine and the first hairpin loop containing the QxVxG motif. These two domains, together with the C-terminal PW sequence, make up the 'wedge' in the cystatin 1 model, shown by crystallography in cystatin B and chicken cystatin to interact directly with the active-site cleft of target C1 proteases [22,23]. These results suggest that diversifying selection could be acting on viral cystatins to modify the inhibitor's sites of interaction with host target proteases, which could translate into an increased or reduced affinity towards these enzymes. Interestingly, modifications in inhibitor affinity have been reported in engineered cystatin proteins carrying deletions or mutations in the N-terminal segment or the first hairpin loop [27,39-41]. In chicken cystatin, the removal of the residues preceding the conserved Glycine leads to a 5000-fold decrease in affinity towards papain [39]. Furthermore, a site-directed mutagenesis approach used to pin-point which residues contribute the most to target enzyme affinity in human cystatin C revealed that the -1 residue (with respect to Glycine) is responsible for the major part of this affinity [40]. In PDV cystatins it is noteworthy to stress that the equivalent site (corresponding to Lysine 5 preceding the conserved Glycine 6 in CcBV cystatin 1) is under positive selection. This suggests that PDV cystatins have evolved under diversifying selection possibly to produce inhibitors of varying affinity for caterpillar proteases, just as cystatin C laboratory-engineered mutants have been developed that have discriminating affinities for mammalian cysteine proteases [42]. We can predict that the other sites under positive selection in the N-terminal region of viral cystatins are also likely to have an influence on the interaction with proteases. Indeed, a comparison of the positions of positively selected sites of PDV cystatins and plant cystatins revealed that two of these sites are in equivalent positions with respect to the conserved Glycine residue in both sets of inhibitors (positions -1, +3). Furthermore, in plant cystatins, independent mutations in these sites lead to variations in inhibitory activity towards papain and cathepsin B [27,43].

Two positively selected sites have also been identified in the first hairpin loop of PDV cystatins including the central valine of the QxVxG motif. These sites, corresponding to Valine 52 and Alanine 53 in cystatin 1, are inside this region with one affecting the central Valine. However, this central site is not absolutely conserved in all cystatins. In the chicken egg white cystatin the hairpin loop motif is QLVSG and an increase in binding affinity to cysteine proteinases was obtained when this motif was mutagenized to QVVAG [44] indicating that variation in central residues of this loop affects binding with target proteases.

In summary, the majority of positively selected sites identified in PDV cystatins are located in the vicinity of the two inhibitory sites analysed in this study. Furthermore, these sites affected by positive selection have been shown experimentally in other cystatins to be important for affinity with target proteases. Taken together these results suggest that positive selection is acting presumably to modulate viral cystatin affinity for caterpillar protease targets.

It will now be interesting to determine what could be the role of the positively selected sites which are more distant from the cystatin inhibitor sites (Lysine 20, Arginine 31, Phenylalanine 58, Asparagine 60 and Leucine 70 in cystatin 1). Phenylalanine 58 and Asparagine 60 may still be influencing the L1 loop at position 50–54. Leucine 70 is located near the disulphide bond and variations in this position may affect the structure of the protein. These sites could also be unmasking new sites of interactions with proteases, indeed in chicken cystatin it was suggested that other regions or sites of the protein could be important for the strong interaction with the cysteine protease cathepsin L [44].

### Scenario for cystatin gene evolution

The strong selective pressure observed emphasizes the important role of cystatins in the host-parasitoid interaction. These results suggest that cystatins have to continuously evolve in order to adapt to their target in the host caterpillar. Given the potential pathogenic role of viral cystatins and also the probable involvement of cysteine proteases in insect immunity [45], these results can be interpreted by integrating cystatins in a coevolutionary context. Nevertheless, this diversifying evolutionary pattern could also be explained by wasp host switches and the subsequent necessity for cystatins to evolve rapidly to respond to new biochemical targets.

Our analysis reveals the existence of two viral cystatin forms which display different evolutionary patterns with regards to wasp evolution. In more classical non-obligate mutualist associations, horizontal gene transfer can explain incongruences between host and symbiont phylogenies. However, in this case, virus and wasp have been in a long and stable relationship for more than 100 million years [46] and artificial infection of wasps by PDV is not possible. Therefore, we propose, as our analyses strongly suggest, that adaptive constraints have contributed to the different evolutionary patterns observed in the two *cystatin *forms.

Moreover, for both of these two forms duplication events occurred independently in the different *Cotesia *bracoviruses studied and are fixed by positive selection which is also responsible of the ensuing divergence of *cystatin *copies.

Interestingly, Francino [28] proposed in the 'radiation adaptive model' that duplications are fixed to their selective advantage and that gene copies evolve under natural selection before new functions appear. This mode of evolution, particularly for functional genes, could be a response to specific environmental pressures such as new biochemical niches. Therefore, the particular evolution of the *cystatin *gene family could be a response to particular cystatin targets in a specific host-parasitoid system.

## Conclusion

Unravelling the molecular evolution of proteins can lead to a better understanding of their function. For the first time in a host-parasite interaction system, we have shown that viral *cystatins *are subject to strong positive selection. Three-dimensional modelling of a viral cystatin revealed that most of the positively selected residues are in the vicinity of the inhibitory active sites, suggesting that adaptive selection acted to improve the inhibitory activity of viral cystatins. Furthermore two different *cystatin *forms have been identified, each of them evolving under different selective constraints probably imposed by different host cysteine proteases.

In order to better explain *cystatin *gene family evolution, we have to consider the host range of each wasp species studied. For this purpose, studying the Melitaeini-*Cotesia *system appears clearly adapted since their ecology in terms of host range is well characterized [47]. Such a study would provide a precise definition of the potential coevolutionary processes involved between viral *cystatins *and host cysteine proteases.

## Methods

### Wasp specimens

*Cystatin *genes were isolated from nine viruses associated with the following *Cotesia *species: *C. congregata*, *C. flavipes*, *C. chilonis*, *C. melanoscela*, *C. vestalis*, *C. rubecula*, *C. sesamiae*, *C. kariyai *and *C. glomerata *(Table 2). These species provide a good representation of *Cotesia *species diversity based on the *Cotesia *phylogeny [29].

**Table 2:** Wasp samples and primers used for cystatin gene amplification

Wasp species	Location	Collections	Primers	Species abbreviations
*C. congregata*	Lab reared	Drezen, J.M (Fr)	Cyst15/Cyst93	*CcBV*
*C. chilonis*	Lab reared	Wiedenmann,. R (USA)	Cyst15/Cyst93	*CchBV*
*C. flavipes*	Kenya	Dupas, S (Fr)	Cyst15/Cyst93	*CfBV*
*C. glomerata*	Lab reared	Vet, L (NL)	Cyst15/Cyst93	*CgBV*
*C. kariyai*	Japan	Tanaka, T (J)	Cyst15/Cyst103	*CkBV*
*C. melanoscela*	France	Villemant, C (Fr)	Cyst15/Cyst93	*CmBV*
*C. vestalis*	Benin	Guilloux, T (Fr)	Cyst15/Cyst93	*CvBV*
*C. rubecula*	Lab reared	Smid, H (NL)	Cyst15/Cyst103	*CrBV*
*C. sesamiae*	Kenya	Dupas, S (Fr)	Cyst15/Cyst103	*CsBV*

### DNA extraction, amplification, cloning and sequencing

DNA extractions were performed using the Chelex method from a whole individual wasp. Wasp tissues were disrupted in a 5% Chelex solution including proteinase K (0.12 mg/ml). Three primers for *cystatin *gene amplification were designed based on an alignment of the three *cystatin *genes from *C. congregata *bracovirus [EMBL: AJ632321] and one *cystatin *gene from *Glyptapanteles indiensis *bracovirus [GenBank: AC191960]; one forward primer Cyst15 5'-ATGGGCAAGGAATATCGAGTG-3' and two reverse primers Cyst93 5'-GTAAGGACAGTTTTTATCTAG-3', Cyst103 5'-GTAAGGACGACTTTTATCTAG-3'. The amplified product is composed of 279 nucleotides and encodes a 93 amino acid sequence containing the first two conserved domains of cystatins. Polymerase chain reaction (PCR) amplification was performed in a 50 μl volume containing 1× Taq buffer, 3 mM of MgCl_2_, 2.5 mM of dNTP, 0.3 μl Taq polymerase (Goldstar, Eurogentec) and 50 pmol of each primer. Goldstar polymerase displays a very good fidelity of one error every 5 × 10^-5^bases. PCR conditions consisted of an initial denaturation step at 94°C for 2 minutes followed by 30 cycles of a denaturation step at 94°C for 45 seconds, an annealing step at 45°C for 1 minute, a polymerization step at 72°C for 45 seconds and final elongation at 72°C for 10 minutes. PCR products were cloned into the pDrive-cloning vector (Qiagen cloning kit). For each species, 12 positive clones were sequenced in order to isolate all the *cystatin *gene copies and to obtain a minimum of two identical clones per sequence. Only CcBV21L and CcBV21I correspond to unique sequences. However, excluding these sequences from the data set does not change the results of the analysis on positively selected sites. Cloned inserts were sequenced in both directions using the Big Dye^R ^Terminator v3.1 cycle sequencing kit and the sequenced products were analysed using a capillary DNA sequencer (ABI PRISM 3100).

### Sequence analysis and phylogeny

*Cystatin *sequences obtained and sequences already available from viral genome sequencing (CcBVcyst1, CcBVcyst2 and CcBVcyst3) were aligned using ClustalW implemented in Bioedit version 5.06 [48]. We estimated the intraspecies and interspecies *cystatin *gene divergence using MEGA ver3.1 [49]. Divergence was calculated by a pairwise distance under the Kimura two-parameter substitution model.

Recombination can mislead phylogenetic estimation and positive selection analysis. In order to avoid this bias we tested the *cystatin *gene family for recombination using a genetic algorithm recombination detection (GARD) implemented in Hyphy [50].

The program MrModeltest ver2.2 [51] was used to determine the appropriate model of DNA substitution by the hierarchical likelihood ratio test (hLRTs). Phylogenetic trees were obtained by maximum likelihood using PHYML program [52] and by Bayesian inference in Mr Bayes 3.12 [53]. Modeltest chose the Kimura 80 model with a gamma distribution of parameter shape α = 0.7875, a transition/transversion ratio of 1.12 and a proportion of invariables sites equal to zero. These parameter estimations were used as initial parameter values for maximum likelihood and Bayesian inference. The topology and branch length estimation by maximum likelihood was repeated 1000 times and for Bayesian analysis we performed 1000000 generations until the standard deviation was below 0.01.

### Positive selection among sites

All of the analyses on the rate of protein evolution among taxa and tests of positive selection were conducted using the codeml program in the PAML package v3.14 [54]. Pairwise estimates of the number of nonsynonymous substitutions per synonymous site (*d*_*N*_) and the number of synonymous sites (*d*_*S*_) were calculated using maximum likelihood [55].

To test for evidence of positively selected sites, we performed different models allowing evolutionary rates (ω = *d*_*N*_/*d*_*S*_) to vary across codon sites (models M0, M3, M8A and M8) [56]. Model M0 (one ratio model) assumes that all branches in the phylogeny and all sites have the same ω. Model M3 classifies sites in the sequence into three discrete classes with ω estimated from the data [56]. M8A assumes a β-distribution of the *d*_*N*_/*d*_*S *_ratio constrained to lie between 0 and 1.0 and adds to the β-distribution a point mass at ω = 1 (see [57]) whereas the selection model M8 permits one additional *d*_*N*_/*d*_*S *_ratio to be above 1. Nested models (that is, M0 versus M3 and M8A versus M8; nonpositively selected versus positively selected models) were compared using the LRT: 2X the log-likelihood difference between the two models can be compared to a χ^2 ^distribution, with the number of degrees of freedom equal to the difference between the two models. Codon sites under positive selection were identified using the Bayes empirical Bayes (BEB) calculation of posterior probability for site classes [58] that analyses the sites under positive selection identified by the selective models. The numbers of substitutions between *cystatin *genes were counted using the 'codeml' program in the PAML package [59], with the F1X4 model of codon frequencies. Four sequences containing a stop codon were eliminated from the analysis. Each analysis was repeated 10 times with different initial ω values to avoid problems of multiple local optima.

### Positive selection among lineages

To test for evidence of positive selection among sites but also among lineages we performed a branch-site analysis using the codeml program in the PAML package v3.14. In this analysis, the branches under positive selection are called 'foreground' branches and all other branches are called 'background' branches. Sites changing in the foreground lineage are permitted to have ω > 1. Yang and Nielsen [60] implemented two versions of branch-site models called MA and MB. In MA, ω_0 _is estimated from the data under the constraint 0 < ω_0 _< 1; hence, positive selection is permitted only in the foreground branch. This model is compared with model M1a. In MB, ω_0 _and ω_1 _are free parameters. Thus, some sites evolve by positive selection across the entire phylogeny, whereas other sites evolve by positive selection in the foreground branch only. MB is compared with M3. The parameters used to perform this analysis are the same as those used in the site analysis.

The branch-site analysis was used to gain information on the possibility of different evolutionary constraints in different lineages. A problem with this method is that it assumes an *a priori *hypothesis. Indeed we have to specify foreground and background lineages with no knowledge of lineage history or of the type of substitutions that occur.

To determine the precise lineage analysis we used a local codon model implemented in HyPhy [50] able to estimate nonsynonymous and synonymous substitutions per site for each branch. This analysis informs us about the kind of substitutions that occurred during *cystatin *lineages divergence.

In addition we used a naive approach to detect branches specifically under positive selection in the tree. The basic principle of this method is to assign each branch of a phylogenetic tree to a particular ω class. Different models assigning branches into different ω classes were tested and compared using the Akaike information criterion (AIC_c_). To search the space of possible models HyPhy employs a Ga that measures the fitness of each model by its AIC_c _score. Ga-branch analysis enables the assignment of lineages in a phylogeny to a fixed number of different classes of ω, thus allowing variable selection pressure without *a priori *specification of particular lineages. Ga-branch analysis as most molecular evolution programs is computationally challenging and imposed that the number of sample sequences be reduced to 25. We therefore removed from our sample all nearly identical sequences and pseudogenes. The evolutionary codon model used for this analysis was determined from the AnalyzecodonData implemented in the Hyphy package.

### Structure prediction and model building

The available structure of chicken egg white cystatin (pdb code 1cew) and human cystatin D (pdb code 1roa) were used as templates [22,30]. The sequence of mature CcBV cystatin1 shares 28% and 24% identity with chicken egg white cystatin [Swissprot: P81061] and human cystatin D [Swissprot: P28325], respectively. Despite the relatively modest level of sequence identity, a reasonable alignment could be made. In particular, the 'wedge' region containing the conserved QxVxG motif could be readily aligned. CcBV cystatin 1 corresponds to a type 2 cystatin, which has two conserved disulfide bridges. For the inter-beta-strand disulfide bond, the sequence alignment and template structure place the Cys residues within disulfide bonding distance. The second pair of Cys residues in the initial model were too distant to form a disulfide bond, and had to be brought closer together through energy refinement. The homology modelling was carried out using the program COMPOSER in SYBYL 7.3 (Tripos Inc, St Louis, MO) on residues 6 to 108 of the mature protein. Three structurally conserved regions (SCRs) were used to build an initial model of CcBV cystatin 1, with three deletions and no insertion relative to the template.

### Molecular model refinement and MD simulations

Structural refinement of the complex was performed by stepwise energy minimization in Sybyl using the AMBER all atom force field [61] to a gradient of 0.05 kcal/mol/Å. First, only the side chains of the SCRs were energy-minimized, followed by energy minimization of the entire structure. The energy-minimized model was then used as the starting point for MD simulations using the AMBER ff03 force field in the AMBER 9 suite of programs [62]. The protein was solvated in a truncated octahedron TIP3P water box [63]. The distance between the wall of the box and the closest atom of the solute was 12.0 Å, and the closest distance between the solute and solvent atoms was 0.8 Å. Counterions (Cl^-^) were added to maintain electroneutrality of the system. The solvated system was energy-minimized with harmonic restraints of 10 kcal mol^-1 ^Å^-2 ^on all solute atoms, followed by heating from 100 to 300 K over 25 ps in the canonical ensemble (NVT). Then, the solvent density was adjusted by running a 25 ps isothermal isobaric ensemble (NPT) simulation under 1 atm pressure. The harmonic restraints were then gradually reduced to zero with four rounds of 25 ps NPT simulations. After an additional 25 ps simulation, a 10 ns production NPT run was carried out with snapshots collected every 1 ps. For all simulations, a 2 fs time-step and 9 Å nonbonded cutoff were used. The particle mesh Ewald (PME) method was used to treat long-range electrostatic interactions [64], and bond lengths involving bonds to hydrogen atoms were constrained by SHAKE [65].

## Authors' contributions

CS participated in the data acquisition and performed the sequence alignment, the phylogenetic and selection analysis and wrote the main draft of the manuscript. SC performed the 3D structure modelling and participated in drafting the manuscript. SP was involved in the main acquisition of data and participated in the critical revision of the manuscript. SD participated in selection analysis and in intellectual revision of the manuscript. JL participated in experimental discussions and sequence acquisitions. EOP was involved in the cystatin 3D structure modelization and in intellectual discussions. JMD was involved in obtaining funding, in project conception and in critical revision of the manuscript. EH was responsible for the project conception and of the experiments and was actively involved in drafting the manuscript. All authors have given their final approval to the version to be published.

## Supplementary Material

Additional file 1 Species names and accession numbers of cystatin sequences. Species names, clone numbers and corresponding accession number. Click here for file

Additional file 2 Alignment of cystatin nucleotide sequences. Sequence name, alignment and consensus sequence. Click here for file

Additional file 3 Alignment of cystatin protein sequences. Sequence name, alignment and consensus sequence. Click here for file
