# Supplementary material for: Viral cystatin evolution and three-dimensional structure modelling: A case of directional selection acting on a viral protein involved in a host-parasitoid interaction
Source: BMC Biol. 2008 Sep 10;6:38. doi: 10.1186/1741-7007-6-38 (PMC2553070; doi:10.1186/1741-7007-6-38)
Supplement: Additional file 1 — Species names and accession numbers of cystatin sequences. Species names, clone numbers and corresponding accession number. [file 1741-7007-6-38-S1.doc]

Additional File 1. Species name and Accession number of cystatin sequences

| Species name | Clone number | Abreviations | Accession number |
| --- | --- | --- | --- |
| Cotesia congregata Bracovirus | Clone: 1 | CcBV1 | EU493286 |
| Cotesia congregata Bracovirus | Clone: 2 | CcBV2 | EU493287 |
| Cotesia congregata Bracovirus | Clone: 21I | CcBV21I | EU493288 |
| Cotesia congregata Bracovirus | Clone: 21L | CcBV21L | EU493289 |
| Cotesia congregata Bracovirus | Clone: 3 | CcBV3 | EU493290 |
| Cotesia congregata Bracovirus | Clone: 6 | CcBV6 | EU493291 |
| Cotesia chilonis Bracovirus | Clone: 10 | CchBV10 | EU493292 |
| Cotesia chilonis Bracovirus | Clone: 7 | CchBV7 | EU493293 |
| Cotesia chilonis Bracovirus | Clone: 4 | CchBV4 | EU493294 |
| Cotesia chilonis Bracovirus | Clone: 3 | CchBV3 | EU493295 |
| Cotesia chilonis Bracovirus | Clone: 2 | CchBV2 | EU493296 |
| Cotesia flavipes Bracovirus | Clone: 9 | CfBV9 | EU493297 |
| Cotesia flavipes Bracovirus | Clone: 7 | CfBV7 | EU493298 |
| Cotesia flavipes Bracovirus | Clone: D | CfBVD | EU493299 |
| Cotesia flavipes Bracovirus | Clone: 8 | CfBV8 | EU493300 |
| Cotesia flavipes Bracovirus | Clone: F | CfBVF | EU493301 |
| Cotesia flavipes Bracovirus | Clone: 5 | CfBV5 | EU493302 |
| Cotesia melanoscela Bracovirus | Clone: 10 | CmBV10 | EU493303 |
| Cotesia melanoscela Bracovirus | Clone: 2 | CmBV2 | EU493304 |
| Cotesia melanoscela Bracovirus | Clone: 3 | CmBV3 | EU493305 |
| Cotesia melanoscela Bracovirus | Clone: 9 | CmBV9 | EU493306 |
| Cotesia melanoscela Bracovirus | Clone: 1 | CmBV1 | EU493307 |
| Cotesia vestalis Bracovirus | Clone: 5 | CvBV5 | EU493308 |
| Cotesia vestalis Bracovirus | Clone: 6 | CvBV6 | EU493309 |
| Cotesia vestalis Bracovirus | Clone: 2 | CvBV2 | EU493310 |
| Cotesia sesamiae Bracovirus | Clone: 2 | CsBV2 | EU493311 |
| Cotesia sesamiae Bracovirus | Clone: 1 | CsBV1 | EU493312 |
| Cotesia sesamiae Bracovirus | Clone: 3 | CsBV3 | EU493313 |
| Cotesia sesamiae Bracovirus | Clone: 4 | CsBV4 | EU493314 |
| Cotesia sesamiae Bracovirus | Clone: 6 | CsBV6 | EU493315 |
| Cotesia rubecula Bracovirus | Clone: 6 | CrBV6 | EU493316 |
| Cotesia rubecula Bracovirus | Clone: 3 | CrBV3 | EU493317 |
| Cotesia rubecula Bracovirus | Clone: 4 | CrBV4 | EU493318 |
| Cotesia rubecula Bracovirus | Clone: 5 | CrBV5 | EU493319 |
| Cotesia rubecula Bracovirus | Clone: 2 | CrBV2 | EU493320 |
| Cotesia rubecula Bracovirus | Clone: 1 | CrBV1 | EU493321 |
| Cotesia kariyai Bracovirus | Clone: 11 | CkBV11 | EU493322 |
| Cotesia kariyai Bracovirus | Clone: 9 | CkBV9 | EU493323 |
| Cotesia kariyai Bracovirus | Clone: 10 | CkBV10 | EU493324 |
| Cotesia kariyai Bracovirus | Clone: 12 | CkBV12 | EU493325 |
| Cotesia kariyai Bracovirus | Clone: 5 | CkBV5 | EU493326 |
| Cotesia glomerata Bracovirus | Clone: 12 | CgBV12 | EU493327 |
| Cotesia glomerata Bracovirus | Clone: G1 | CgBVG1 | EU493328 |
| Cotesia glomerata Bracovirus | Clone: 11 | CgBV11 | EU493329 |
| Cotesia glomerata Bracovirus | Clone: G7 | CgBVG7 | EU493330 |
