# Supplementary figures and images for: Viral cystatin evolution and three-dimensional structure modelling: A case of directional selection acting on a viral protein involved in a host-parasitoid interaction
Source: BMC Biol. 2008 Sep 10;6:38. doi: 10.1186/1741-7007-6-38 (PMC2553070; doi:10.1186/1741-7007-6-38)

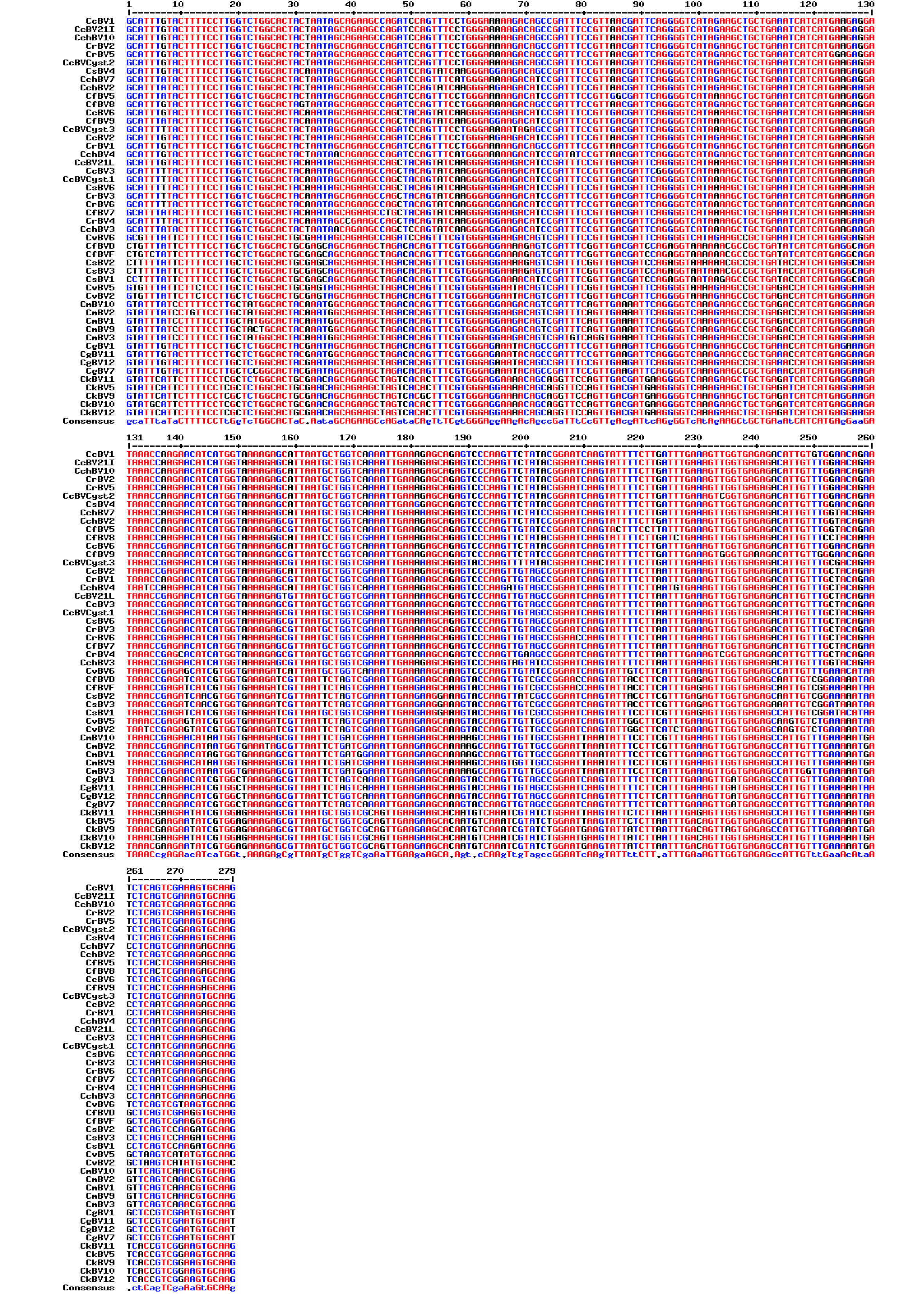

Supplement: Additional file 2 — Alignment of cystatin nucleotide sequences. Sequence name, alignment and consensus sequence. [file 1741-7007-6-38-S2.tiff]

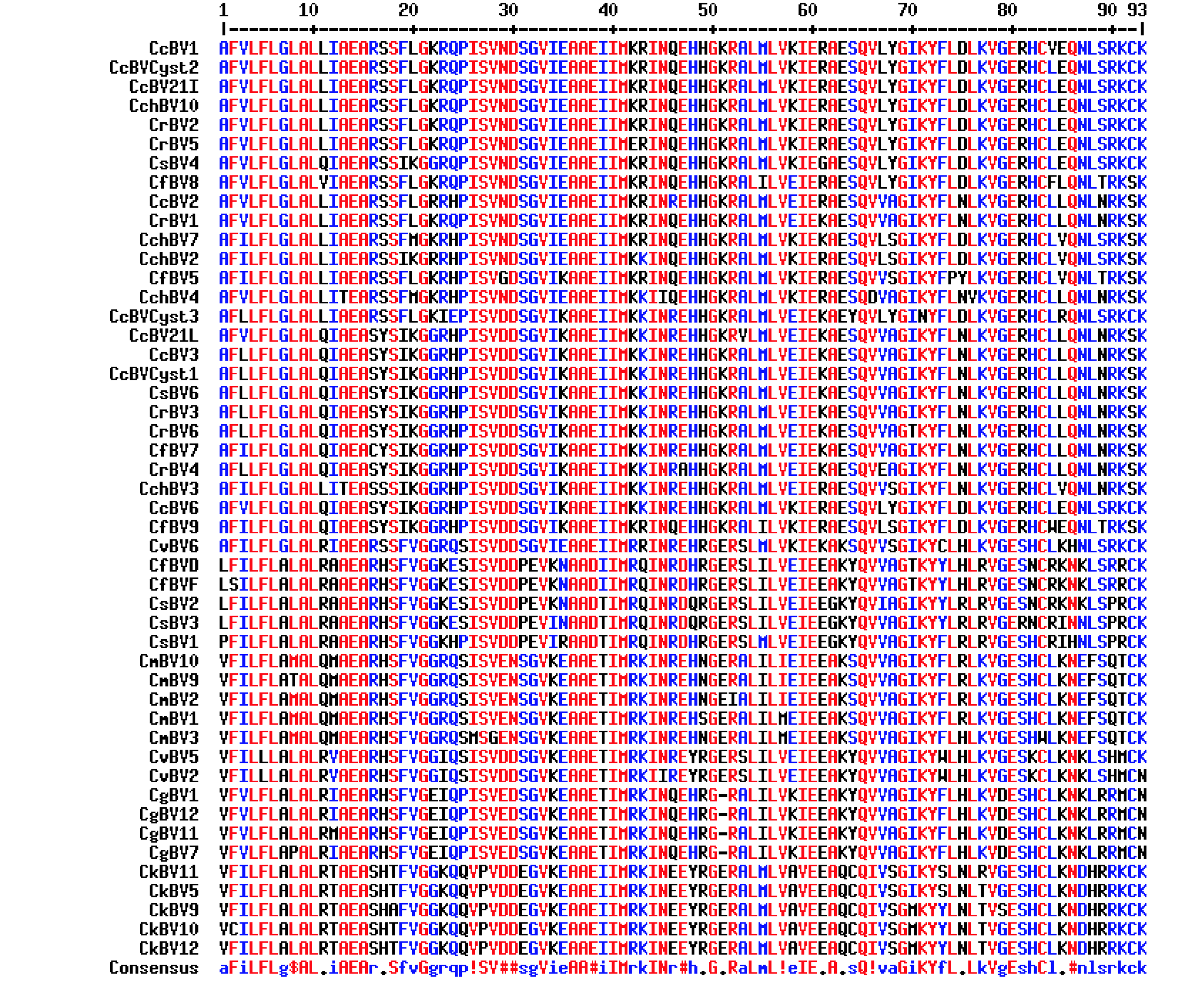

Supplement: Additional file 3 — Alignment of cystatin protein sequences. Sequence name, alignment and consensus sequence. [file 1741-7007-6-38-S3.tiff]
